# Resilience of lung grafts to warm ischemia: assessment by *ex vivo* perfusion using functional and molecular analysis 

**DOI:** 10.3389/ti.2026.16314

**Published:** 2026-06-24

**Authors:** Antoine Premachandra, Florentina Pascale, Chloé Mimbimi, Sébastien Jacqmin, Luc Jouneau, Christophe Richard, Valérie Gelin, Claudia Bevilacqua, Jérôme Lecardonnel, Julie Rivière, Nicolas Bertho, Delphyne Descamps, Matthieu Glorion, Morgan Le Guen, Isabelle Schwartz-cornil, Edouard Sage

**Affiliations:** 1 Université Paris-Saclay, INRAE, UVSQ, VIM, Jouy en Josas, France; 2 ERL INSERM U1369, Jouy en Josas, France; 3 Department of Anesthesiology, Foch Hospital, Suresnes, France; 4 Department of Thoracic Surgery and Lung Transplantation, Foch Hospital, Suresnes, France; 5 Université Paris-Saclay, INRAE, UVSQ, BREED, Jouy en Josas, France; 6 Université Paris-Saclay, INRAE, AgroParisTech, GABI, @Bridge, Jouy en Josas, France; 7 Université Paris-Saclay, INRAE, AgroParisTech, Micalis Institute, Jouy en Josas, France; 8 Oniris, INRAE, Bioepar, Nantes, France

**Keywords:** ischemia-reperfusion injuries, lung, pig model, transplantation, warm ischemia

## Abstract

To address the shortage of donor lungs, donation after circulatory death with extended warm ischemia (WI) is increasingly used. Normothermic *ex vivo* lung perfusion (EVLP) allows assessment and rehabilitation of WI lungs. However, the safety limits of WI duration remain unclear due to conflicting preclinical data and variations in clinical protocols across countries. Using a clinically-relevant pig model, we compared paired right (control) and left (WI) lungs from five donors. WI lungs experienced 2-h WI followed by 1-h cold preservation and 6-h EVLP, while control lungs underwent the same protocol without WI. Initial 2-h WI impaired compliance by 32% and increased vascular resistance by 25%, but both parameters normalized during EVLP. Oxidative and mitochondrial stress markers, cytokine release, histological injury, and edema showed no significant difference between control and WI lungs. During EVLP, both control and WI lungs exhibited similar transcriptomic responses in terms of the number of regulated genes (69/96) and their expression levels. Overall, EVLP reversed initial WI-associated functional impairments and led to convergent molecular profiles in WI and control lungs. These findings support the possibility of extending acceptable WI duration thresholds in lung transplantation.

## Graphical Abstract

**Figure:**
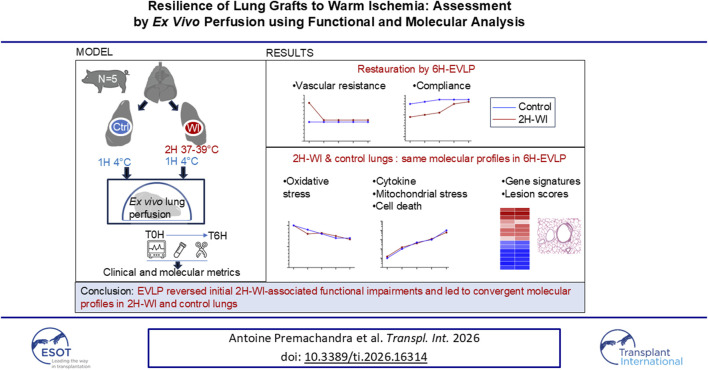

## Introduction

Due to the persistent shortage of donor lungs, donation after circulatory death (DCD) and prolonged warm ischemia (WI) are increasingly tolerated for transplantation, despite elevated risk of ischemia-reperfusion injury (IRI) [1]. To mitigate this risk, normothermic *ex vivo* lung perfusion (EVLP) is used to assess and rehabilitate these grafts. In countries with formal legal frameworks (e.g., France and Italy), EVLP is mandatory for DCD lungs, whereas in other countries, its use is guided by clinical judgment or institutional protocols. There is no consensus on the safe duration of WI after DCD. Clinical limits vary and are based on inconsistent preclinical and clinical results. Indeed, some studies reported that WI beyond 60 min exacerbated inflammatory responses and impaired respiratory function during EVLP [2–4]. In contrast, retrospective clinical analyses have shown that DCD lungs with a mean WI duration of up to 208 min exhibited inflammatory profiles during EVLP and early post-transplant outcomes similar to those of marginal lungs from brain-dead donors [5]. Interpretation of preclinical data is complicated by inconsistencies in EVLP protocols, particularly due to the variable inclusion of erythrocytes and a lack of corticosteroids, the latter being standardly used in clinical settings. In this context, national guidelines differ widely in their maximum permitted WI durations: 90 min in France [6] and Australia [7], 120 min in the UK [8], 150 min in Spain [9], and up to 180 min in Italy [5].

We hypothesized that lungs subjected to an extended 2-h WI period may be rehabilitated by EVLP. The objective of this report was to assess the impact of a 2-h WI period on lung responses to a clinically EVLP relevant protocol (i.e., the Toronto technique with corticosteroids), in a porcine lung model, widely recognized as the most translationally relevant model. We performed a comprehensive comparison of paired right and left lungs undergoing EVLP under control conditions (control lungs, right) and after a 2-h WI (WI lungs, left) using a broad range of metrics.

## Materials and Methods

### Ethics

The experiments were performed in compliance with the EU guidelines and the French regulations (DIRECTIVE 2010/63/EU, 2010; Code rural, 2018; Décret n°2013-118, 2013). The procedures received authorization of the Ministry of Higher Education and Research and the experimental protocols were approved by the COMETHEA ethic committee under the number APAFIS# DAP2022120510144946 v3. The surgery was carried out at the Medical Imaging in Animal platform (accreditation B78-322-2, *doi.org/10.15454/1.5572348210007727E12*).

### Animals, and EVLP procedures

Lungs were harvested from five Large-White x Landrace x Pietrain pigs (48–55 kg). Animals were anesthetized with intramuscular ketamine (20 mg·kg^-1^, Imalgene®) and xylazine (2 mg·kg^-1^, Rompun®). Orotracheal intubation was performed after deepening anesthesia with 3%–5% isoflurane using mask, and intravenous injection of fentanyl (20 μg·kg^-1^) and diazepam (0.1 mg·kg^-1^). Anesthesia was maintained with 2%–3% inhaled isoflurane and continuous propofol infusion at 5 mg·kg^-1^·h^-1^ (Proposure®). Mechanical ventilation was initiated with a tidal volume (Vt) of 7 mL·kg^-1^, a positive end-expiratory pressure (PEEP) of 5 cmH_2_O, and a respiratory rate of 15 cycles·min^-1^. Upon chest opening, a biopsy from the azygos lobe was harvested (designated as the “Time of death” time point). The animal was then euthanized by aortic clamping and exsanguination following intravenous administration of 25,000 IU of heparin. Euthanasia marked the onset of WI. After inflation, each lung was isolated. The right lung (control group) was immediately flushed with 2 L Perfadex® at 4 °C (XVIVO Perfusion, Gothenburg, Sweden) and stored in the refrigerator at 6 °C for 1 h. The left lung (WI group) remained inflated *in situ* for 2 h, before being similarly flushed and stored at 6 °C for 1 h. Each lung was then connected to an EVLP circuit following the Toronto protocol [10]. The circuit was primed with 1 L of Steen® solution supplemented with 0.5 g methylprednisolone, 0.75 g cefuroxime, and 3750 ui heparin. The lung size differences were taken into account to determine the flow of 16% (left) and 24% (right) of the theoretical cardiac output. The system reached 37 °C after approximately 30 min of perfusion. Ventilation settings were adjusted to a Vt of 3.5 mL/kg for the right lung and 2.5 mL/kg for the left lung, with a fraction of inspired oxygen FiO2 of 0.21, positive and expiratory pressure (PEEP) of 5 cmH_2_O and a respiratory rate of 15 breaths per minute. The perfusate was deoxygenated using a gas mixture containing 8% CO_2_, 86% N_2_O, and 6% O_2_. EVLP was maintained for a total duration of 6 h.

### EVLP monitoring, sample collections

Pulmonary gas exchange capacity was evaluated using PaO_2_ and PaCO_2_ measured in the perfusate at an FiO_2_ of 0.21, using i-STAT cartridges (Abbott, Chicago, USA). Respiratory mechanics and mechanical ventilation were assessed by measuring plateau pressure (Pplat), driving pressure (ΔP = Pplat–PEEP), pulmonary compliance (Vt/ΔP) and recruitment (compliance change during a PEEP test performed at 2 cmH_2_O and 8 cmH_2_O). The pulmonary strain was estimated as stress divided by specific elastance, with stress approximated by Pplat for isolated lungs, and specific elastance estimated in pigs at 6 cmH_2_O [11]. Hemodynamics were evaluated by calculating pulmonary vascular resistance (PVR = [(mPAP – 0)/ECMO flow] × 80). Perfusates were collected at hourly intervals and frozen at −80 °C for lactate deshydrogenase (LDH), mitochondrial DNA and cytokine detection. Lung tissue samples (about 100 mg) were collected using a lung stapler from the azygos lobe just before exsanguination, from the superior lobe at the end of the cold ischemic phase and after 2-h perfusion, and from the inferior lobe after 4- and 6-h perfusion. These samples preserved in RNAlater (Merck, Darmstadt, Germany) or directly frozen in liquid nitrogen and kept at −70 °C. Additionally, a 100 mg lung tissue sample collected at 6 h was fixed in 4% buffered paraformaldehyde for histological analysis, then rinsed after 24 h and stored in 70% ethanol. Bronchoalveolar lavages were performed with 2 × 20 mL cold PBS, and samples were kept frozen at −70 °C.

### Biological dosages and histology

Detailed histology protocols, the commercial kits employed (LDH, cytokine assays, carbonylated proteins, oxidized DNA), and mitochondrial DNA quantification by PCR (ND1 and ND4 genes) are described in Supplementary Material 1.

### Transcriptomic analyses

Total RNA was extracted from lung tissue using TRIzol reagent with bead homogenization (Precellys 24 bead grinder) and purified with the NucleoSpin RNA kit including DNase treatment. RNA quantity and integrity were assessed using the Qubit RNA assay kit and Agilent 2100 Bioanalyzer. Reverse transcription (1.2 µg RNA) was performed using the PrimeScript RT reagent kit. Gene expression was measured using custom TaqMan Array Cards (96 genes, 4 housekeeping genes, Supplementary Material 2) on a QuantStudio 7 Flex Real-Time PCR System. Data were analyzed using the 2^−ΔΔCT^ method normalized to RPS24, RPLP2, and GAPDH; genes with Ct > 32 were excluded. Principal component analysis was performed (mixOmics). Differentially expressed genes (DEGs) versus “Time of death” were identified using paired one-way tests with Benjamini–Hochberg correction (adjusted p < 0.1). Fold changes were calculated relative to “Time of death”. Comparisons between all conditions and the 2-h WI at time zero used paired non-parametric tests with multiple testing correction (adjusted p < 0.2). More details are provided in Supplementary Material 1.

### Statistical analyses

The statistics of gene expression is described in the TAC array design and analysis. For the physiological and biological parameters, a Linear Mixed Model with fixed effects for group, time, and their interaction, and a random intercept for pig subject, was used to analyze repeated measures using R Studio2 v2024.09.0 + 375; the fixed effects were tested with a type III ANOVA. For the *post hoc* comparisons between lung groups, a Tukey test was performed (paired data), using estimated marginal means, and provided adjusted p-values. For histology and percent weight gain, the data were statistically compared using a paired t-test, after checking normal distribution (Shapiro-Wilk). Supplementary Material 3 reports the raw data, means, standard deviation as well as the statistical results (F-val, p-val).

## Results

### The effects of 2-h WI on pig lung functions are corrected by EVLP

**FIGURE 1:**
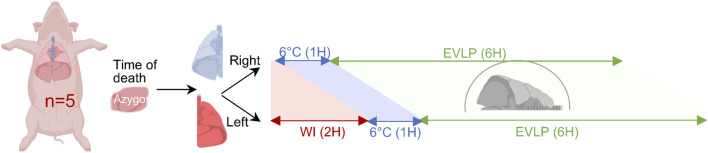
Experimental scheme. Lungs were obtained from 5 pigs. After general anethesia, the azygos lobe was removed and used as a reference lung tissue sample designated as “Time of death” in the whole paper. After subsequent euthanasia by exsanguination, the right and left lungs were separated. The right (Control) was immediately flushed with Perfadex, kept at 6 °C for 1-h, and subjected to EVLP using the Toronto protocol, during 6 h. The left lung (warm ischemia, WI) was placed in the thoracic cavity for 2-h, and then similarly processed as the right lung.

**FIGURE 2:**
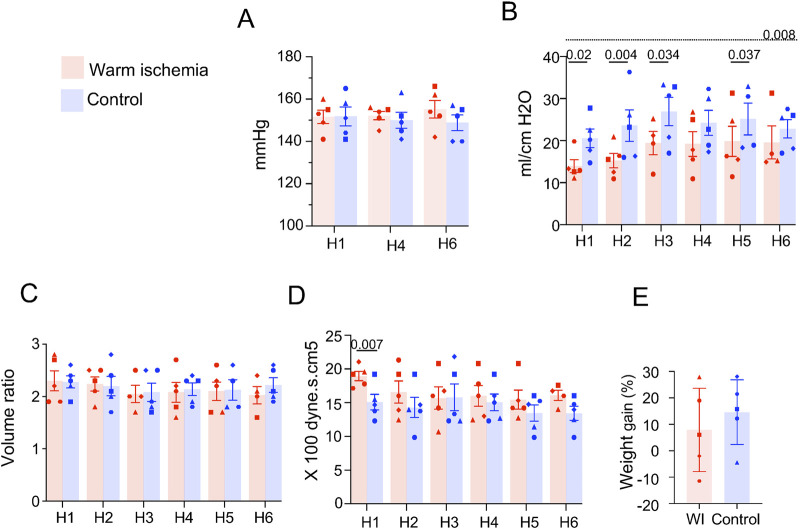
Physiological respiratory parameters. **(A)** PaO2, Partial pressure of oxygen in arterial blood. **(B)** Lung compliance. **(C)** Strain (change in lung volume divided by functional residual capacity). **(D)** Vascular resistance. **(E)** Weight gain at 6H versus 0H (% increase). For **(A–D)**, a linear mixed model with fixed effects for group, time, and their interaction, and a random intercept for pig subject, was used to analyze repeated measures. Type III ANOVA was performed, followed by Tukey-adjusted *post hoc* comparisons using estimated marginal means. Statistical significance is indicated as i) adjusted p-val between groups at specific time points, and ii) as the time effect p-val for both groups over the dashed line. For E, the data were statistically compared using a paired t-test, following a test for assessing the normal distribution of the data (Shapiro-Wilk). Data from the control group are in blue and data from the WI group are in red. Each pig is labelled with the same symbol in all figures (triangle, square, diamond, circle, hexagon).

**FIGURE 3:**
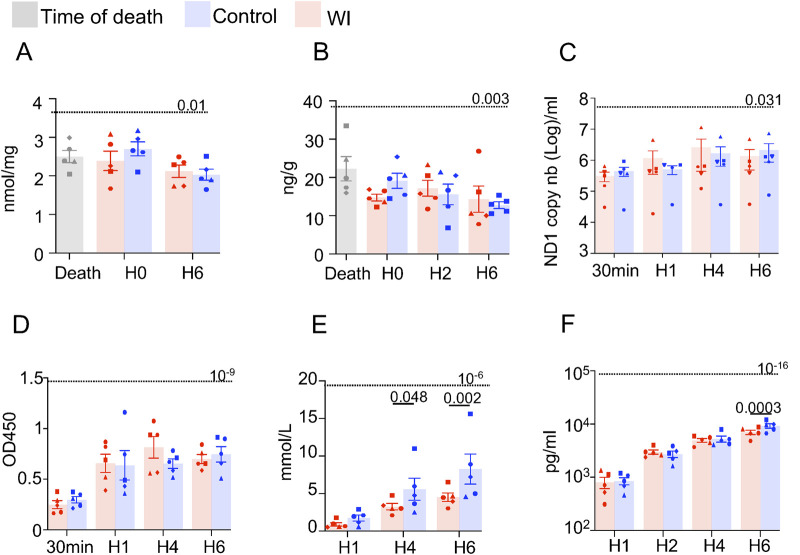
Cellular stress and injury parameters. **(A)** Carbonylated proteins were measured by an ELISA test using frozen lung tissue samples and the results were expressed as ng carbonylated proteins per mg total proteins. **(B)** The 8-hydroxy-2′-deoxyguanosine in DNA was measured by an ELISA test using frozen lung tissue samples and the results were expressed as ng oxidated DNA per Gram of tissue. **(C)** The mitochondrial DNA levels were evaluated by quantifying the pig mitochondrial ND1 gene in perfusion liquids using quantitative PCR. The results are expressed as ND1 gene copy numbers per ml. **(D)** The lactate deshydrogenase (LDH) liberated in the perfusion liquid was measured by a colorimetric assay and OD values minus background are reported. **(E)** Lactate levels were measured with iStat cartridge from fresh perfusion liquid. **(F)** PECAM shedding was measure with a pig luminex discovery assay. A linear mixed model with fixed effects for group, time, and their interaction, and a random intercept for pig subject, was used to analyze repeated measures. Type III ANOVA was performed, followed by Tukey-adjusted *post hoc* comparisons using estimated marginal means. Statistical significance is indicated as adjusted p-val between groups at specific time points, and as the time effect p-val over a dashed line for both groups. Data from the control group are in blue and data from the WI group are in red. Each pig is labelled with the same symbol in all figures (triangle, square, diamond, circle, hexagon).

**FIGURE 4:**
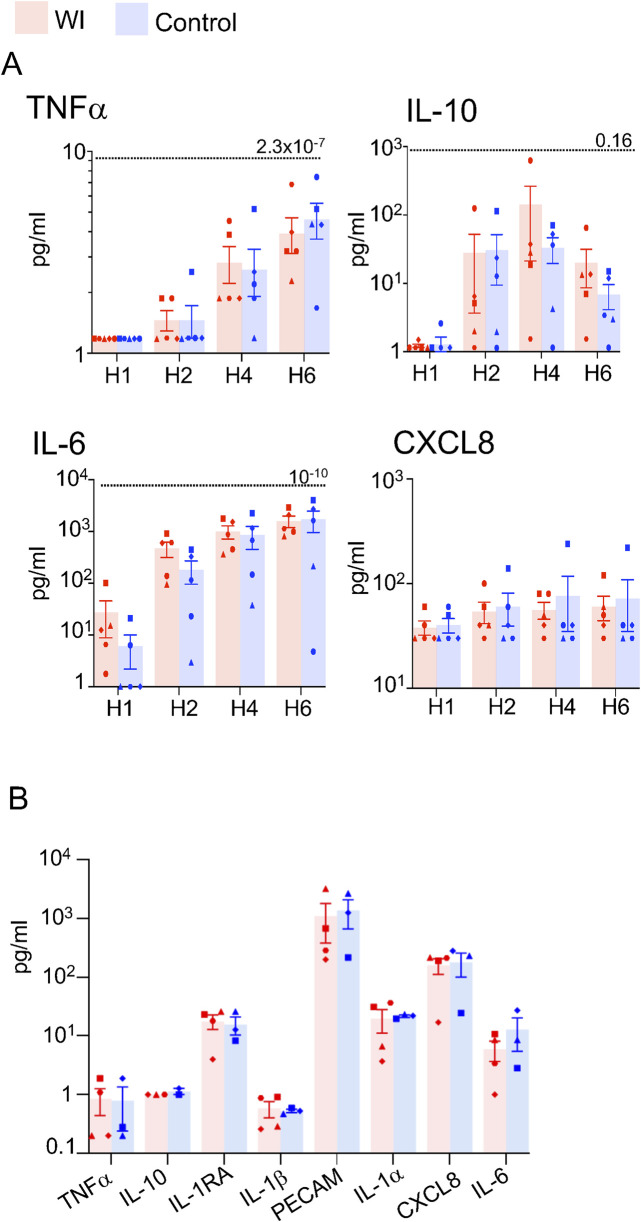
Cytokine release in the perfusion liquid at different timings **(A)** and in the bronchoalveolar lavage at 6 h **(B)**. Cytokine levels were measured with a Porcine Luminex Discovery Assay. In perfusion liquids, IL-1α, IL-1β, IL1RA levels were below the detection threshold and not represented. A linear mixed model with fixed effects for group, time, and their interaction, and a random intercept for pig subject, was used to analyze repeated measures. Type III ANOVA was performed, followed by Tukey-adjusted *post hoc* comparisons using estimated marginal means. Statistical significance is indicated as the time effect p-val over a dashed line for both groups. There was no statistically significant differences between the control and WI groups. Data from the control group are in blue and data from the WI group are in red. Each pig is labelled with the same symbol in all figures (triangle, square, diamond, circle, hexagon).

**FIGURE 5:**
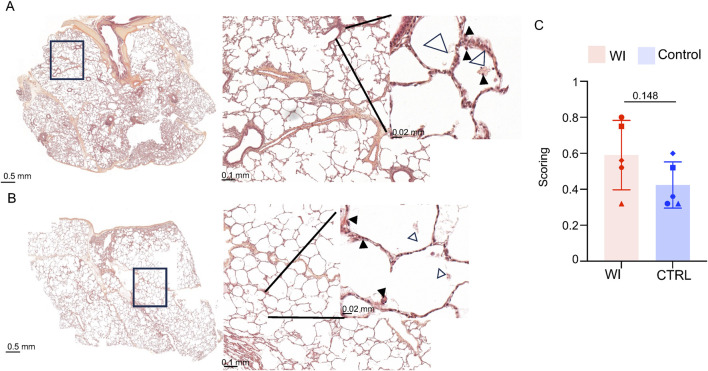
Histological analysis of control and WI lungs after EVLP. In **(A,B)**, representative images of H&E-stained lungs from the control and WI groups following 6H EVLP, at 3 magnifications (X2 left panel, X10 middle panel, and X63 in right panel (magnified field). The black arrows point to granulocytes, and the empty arrows to proteinaceous debris. In **(C)**, the injury scores for each pig of the two groups are reported. The data were statistically compared using a paired t-test, following a test for assessing the normal distribution of the data (Shapiro-Wilk).

Lungs were explanted from five pig donors (see Supplementary Material 1). The azygos lobe was sampled immediately post-mortem (“Time of death”). Each lung block was divided into right and left lungs to allow paired analysis. The right lung (control group) was kept at 6 °C for 1 hour and immediately subjected to 6-h EVLP. The left lung (WI group) remained for 2 h in the thoracic cavity, before undergoing the same cold preservation and EVLP protocol (Figure 1). At the time of surgery, the animals’ body temperatures were within the physiological range (38.5 °C–39 °C) and decreased to 35 °C–37 °C 2 hours after death. During EVLP, oxygenation was maintained in both groups, with PaO_2_ values consistently exceeding 150 mmHg (Figure 2A, see Supplementary Material 3 for the raw data of Figures 2–5). Initial lung compliance was lower in the WI group than in the control group (32% lower, p-val = 0.02), and improved over EVLP time (time effect p-val = 0.008 for both groups, Figure 2B). The strain ratios were below 2.5 and not different between groups (Figure 2C). The initial vascular resistance was higher in the WI than in the control group (25.6% higher, p-val = 0.007), and then the difference became non-significant during EVLP (Figure 2D). At 6 h, weight gain was limited and comparable between groups (percent weight gain: 7.89 ± 11.76 in WI and 14.61 ± 12.23 in control group, Figure 2E). So overall, although 2-h WI initially impaired lung functions (reducing compliance and increasing vascular resistance), these effects were corrected by a 6-h EVLP.

### Extended WI in pig lung has little impact on the IRI response during EVLP

Biological markers associated with ischemia–reperfusion injury were measured during EVLP (Figures 3–5). At baseline (“Time of death”), carbonylated protein levels in lung tissue reached mean values of 2.5 mmol/mg protein (Figure 3A). These levels decreased over time during EVLP (time effect p = 0.01), with no difference between groups at any time point. Similarly to carbonylated protein levels, oxidative DNA levels showed a temporal decrease in both groups (Figure 3B). Mitochondrial DNA concentrations in the perfusate increased over time (time effect p = 0.031), without intergroup differences (Figure 3C). LDH was progressively liberated in the perfusion liquid (time effect, p-val <0.001), without difference between groups (Figure 3D). The metabolic response reflected by lactate production increased over time (p-val in both groups <0.001) and showed higher values in the control group as compared to in the WI group particularly at 6 h (p-val = 0.002, Figure 3E). Finally, the levels of PECAM release also increased over time with higher values in the control group at 6 h (p-val in both groups = 0.0003, Figure 3F). Cytokine concentrations in the perfusate increased over time for TNFα, IL-6, and IL-10, with no differences between groups (Figure 4A). Similar findings were observed in bronchoalveolar lavage samples (Figure 4B). Histological assessment at 6 h showed low injury scores in both groups, with no significant difference (WI: 0.59 ± 0.19 vs. control: 0.42 ± 0.12; p = 0.145; Figures 5A–C).

### Extended WI in pig lung primes but does not escalate the EVLP-associated gene expression

**FIGURE 6:**
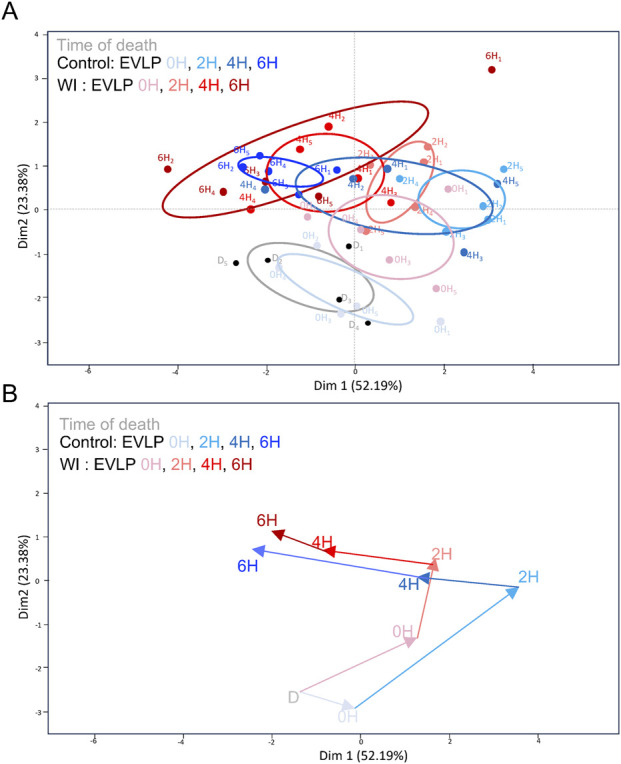
Principal component analysis of the gene expression data at 0, 2, 4 and 6 h. **(A)** The DCT values of 84“EVLP” genes with Ct values >32 from the EVLP lung samples of 5 donor pigs (D1 to D5) were used to generate a principal component analysis with R Studio 2. The first two dimensions represent the highest percent of variance (52.19% and 23.38%). The azygos samples harvested just before death (« Time of death » samples) are shown in grey (D1 to D5), the Control samples are shown in blue (from light blue at 0H to dark blue at 6H)) and the WI samples are shown in red (from light red at 0H to dark dark at 6H), with their respective confidence ellipse. Note that the control ellipse overlaps with the « Time of death » ellipse, the 6H EVLP from the control and WI ellipses overlap together, whereas the 0H WI ellipse lays in between the “Time of death” and 2H EVLP samples. **(B)** The barycenter dynamics is represented by arrows corresponding to each sample group.

We designed a 96 PCR-array using genes previously shown by us and others to be modulated by EVLP ([12–18], Supplementary Material 2). We also incorporated the PDK4 and FKBP5 genes that are upregulated by corticosteroids. Among the 96 genes, 84 genes were retained for analysis based on expression thresholds (Cq < 32; see Methods). Principal component analysis of 2^−ΔΔCT^ values showed overlap between samples obtained at “Time of death” and those from the control group at EVLP initiation (0 hour-EVLP) (Figures 6A,B). During EVLP, transcriptomic profiles from the control and WI groups followed similar trajectories and converged at 6 h. Interestingly, at 0-h EVLP, the WI group samples were located in the EVLP trajectory.

**FIGURE 7:**
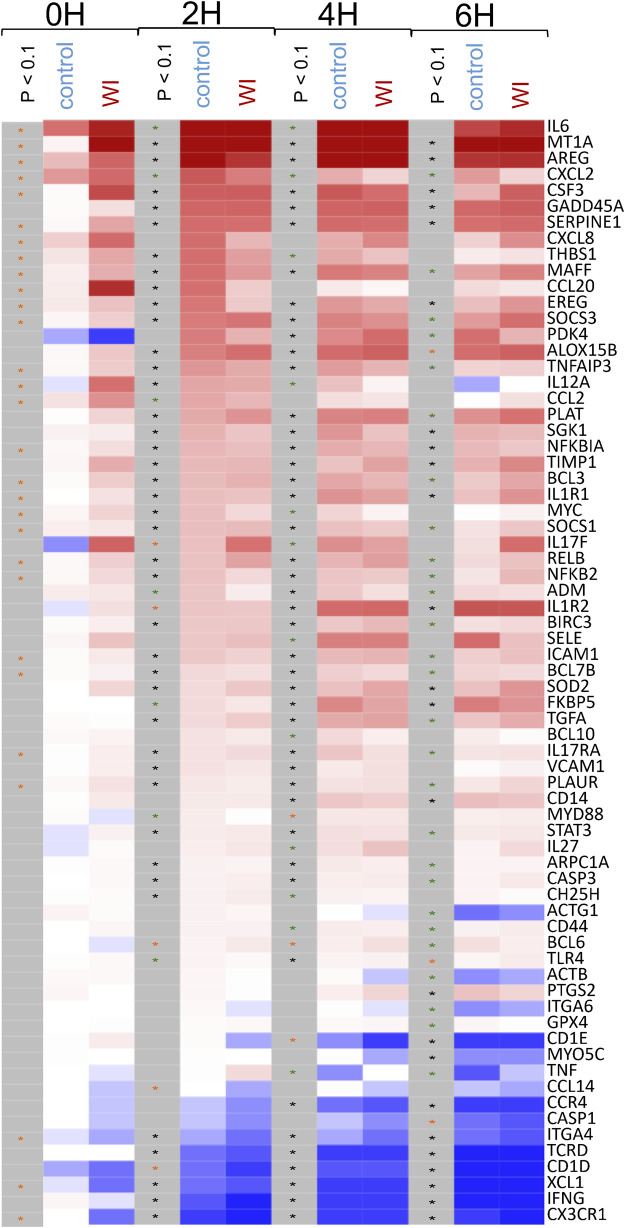
Heat map of the differentially expressed genes versus “Time of death”. Fold changes (FC) in gene expression were calculated at each time point (0H, 2H, 4H, 6H EVLP) relative to the Time of death. Mean FCs were computed per group. Genes showing differential expression (adjusted *p* < 0.1) at any time point compared to “Time of death” were selected for illustration. Black star: genes significantly modulated in both Control and WI groups at a given timing. Orange star: genes significantly modulated only in the WI group. Green star: genes significantly modulated only in the Control group.

For each condition (group and time), a list of differentially expressed genes relative to the “Time of death” (DEGs, p-val <0.1) was established, revealing 69 genes that were differentially expressed at least once (Figure 7, Supplementary Material 4). Among these DEGs, genes related to inflammation (*IL6, CXCL2, CXCL8, CCL2, IL17F, TGFA*), IRI (*CD14, TLR4, MyD88, STAT3*) and oxidative stress (*GADD45, MT1A, SOD2*) were upregulated, whereas genes related to immune activation (such as *CD1E, TCRD, XCL1, IFNG*) and cytoskeleton signaling and cell adhesion/migration) (*ACTG1, ACTB, Myo5C, ITGA4, ITGA6, CX3CR1*) were downmodulated. *PDK4* and *FKBP5* were upregulated only during EVLP, reflecting effective response to corticosteroids. Figure 7 shows that among these 69 DEGS, 29 were significantly modulated by WI *per se* at 0-h EVLP whereas none were found in the control condition at 0-h EVLP. Thereafter during EVLP, there was no significant difference in the gene expression fold changes between the WI and control group at any time point. Furthermore at 2- and 4-h EVLP, the WI and control groups presented a majority of DEGs in common (40 out of 52 DEGs at 2 h, and 42 out of 58 DEGs at 4 h). At 6 h, the WI group showed a high dispersion of the FC values between pig lungs, leading to less DEGs in the WI group than in the control group (51 in the control group, 27 in the WI group, and 23 in common). Finally, when comparing gene expression during EVLP conditions to that of WI lungs at 0-h EVLP, we found that the absolute mean FC of most DEGS (42 out of 69) tended to be higher (p-val <0.2, Supplementary Material 4).

## Discussion

The present study evaluated the impact of 2-h WI on donor lungs using a paired porcine EVLP model. Two-hour WI initially increased vascular resistance and reduced compliance; interestingly, both parameters normalized after 6 h of perfusion. Physical recruitment of atelectasis may also have contributed to the normalization. Compliance and resistance values were consistent with published reports, taking into account known differences between single- and double-lung procedures [16, 19, 20]. Overall, biological measurements obtained during 6-h EVLP did not indicate increased cellular stress or inflammation in the 2-h WI group compared with the control group. Markers of oxidative stress in tissue (protein carbonylation and oxidized DNA), mitochondrial DNA and LDH release, inflammatory cytokines in perfusate and BAL, and PECAM shedding in the perfusate—a marker of endothelial stress—were all comparable between the two groups. Transcriptomic analysis further showed that EVLP induced similar gene expression changes in both groups (compared to “Time of death”), involving pathways related to inflammatory responses, ischemia–reperfusion injury, oxidative stress, cell death, immune activation, cell adhesion/migration, and cytoskeletal signaling, consistent with previous reports [15, 21]. No significant differences in gene expression were detected between the WI and control groups at any EVLP time point. Notably, transcriptomic changes induced by WI alone (at 0-h EVLP) overlapped with the EVLP-driven gene expression trajectory, and profiles converged between groups by the end of perfusion. Taken together, these physiological and molecular findings suggest that lungs subjected to 2-h WI can be effectively recovered by 6-h EVLP, supporting the clinical expansion of WI tolerance thresholds.

As a consequence of the disruption of oxygen supply and impaired ATP production in cells, WI generally induces IRI through ROS production, mitochondrial stress, and changes in energetic metabolism, cell death and the release of inflammatory cytokines [16, 22]. However, the lung appears to show resilience to WI, possibly due to lower metabolic demands and higher oxygen storage capacity, as compared to other organs such as the liver and kidney that withstand less than 30 min WI [5, 23]. For instance, in the pig model, the respiratory functions after transplantation were similar in lung grafts from non-heart-beating donors (with up to 90 min WI) and heart-beating donors [24]. Furthermore, in a clinical study, extension of WI beyond 60 min was not associated with higher mortality nor with worse outcomes following lung transplantation [23]. Indeed, in a retrospective Spanish study, the short and mid-term outcomes were comparable between uncontrolled circulatory death and brain death donors [25].

In contrast with our results, several experimental studies reported that prolonged WI (1–2 h) promoted the inflammatory responses upon EVLP in pig and rat models [2, 4, 26]. A main difference of these studies with our model resides in the use of a high dose of methylprednisolone during EVLP in our case, as done in the clinical settings. Indeed, we found that this treatment induced the genomic response of corticosteroid response genes (PDK4 and FKBP5). This treatment may also explain the reduced expression of genes involved in immune activation, cytoskeleton, and adhesion/migration pathways. However, this is unlikely the case for the *ACT, TCRB,* and *ITGA6* genes that were shown in other systems to be unaffected or even upregulated by dexamethasone [27–29]. The high dose methylprednisolone may also have reduced ROS production, which could account for the low levels of carbonylated proteins and oxidized DNA.

Surprisingly, we observed higher levels of lactate and PECAM in the perfusion fluids of the control group compared with the WI group. One possibility is that the 2-h WI period may have primed lung cells to better tolerate the subsequent EVLP stress. Indeed, for instance, anti-oxidant gene expression pathways were found to be induced by 2-h WI, as reflected by increased levels of *MT1A, GPX4* and *SOD2* transcripts (Figure 7). The anti-oxidant pathway may reduce the glycolytic metabolism in the mitochondria and partially reduce the vascular stress induced by EVLP, resulting in lower levels of lactate and PECAM release.

Our study presents several limitations. The resilience to WI found here in our pig model may not be translatable to other species, and may vary between pig genetic strains [30]. We used inflated lungs during WI; however, outcomes may differ under rhythmic ventilation. Although the sample size was limited to five pigs, the split-lung design enabled paired intra-animal comparisons, providing greater robustness than independent group analyses. Nevertheless, variability within this small cohort may have masked subtle differences between conditions.

Critically, additional experiments with full transplantation following extended WI periods and EVLP should be conducted to better assess functional recovery; indeed, the biological metrics used here likely do not capture the full complexity of tissue repair and recovery. In particular, our analyses focused largely on transcriptomics, which are limited to RNA-level changes. WI followed by EVLP may also additionally affect translational and post-translational processes, metabolism, enzymatic activities, and cell death pathways -including apoptosis, ferroptosis and pyroptosis- which were not assessed here. Notably, no differences in cytokine expression were detected at the protein level between control and WI groups during EVLP. Additional studies using unbiased bulk RNA-seq would strengthen the conclusions or alternatively, may reveal differential expression modulation specific to the 2-h WI group. Extending EVLP beyond 6 h may also uncover delayed biological effects. Finally, while our model focuses on a 2-h WI duration in a controlled experimental setting, the circulatory death in real life involves additional phases of hypoperfusion and inter-organ signaling responses induced by WI. Despite these limitations, our results provide a reference point, i.e., 2-h WI, at which lungs appear amenable to rehabilitation by EVLP.

Donation after circulatory death is highly considered for the future of organ donation. Furthermore, the end-of-life pathways are increasingly linked to protocols of organ donation, therefore, knowledge regarding the impact of WI duration is of highest importance. Future research should systematically extend WI duration followed by EVLP to identify measurable biomarkers defining lung tolerance limits to WI. Prolonged WI duration in experimental models may reveal transcriptomic, metabolic or protein expression patterns during EVLP that diverge from control conditions, signaling impaired recovery. While a ceiling effect in the EVLP response may occur even in control conditions, such models are worth pursuing, as the identification of a non-recovery signature could help determine which WI-exposed lungs are suitable or not for transplantation and may also unravel therapeutic targets.

## Data Availability

The datasets presented in this study can be found in online repositories. The names of the repository/repositories and accession number(s) can be found in the article/Supplementary Material. The PCR array data were deposited on ZENODO under DOI 10.5281/zenodo.18389866.
