# Supplementary material for: Resilience of lung grafts to warm ischemia: assessment by ex vivo perfusion using functional and molecular analysis
Source: Transpl Int. 2026 Jun 24;39:16314. doi: 10.3389/ti.2026.16314 (PMC13341573; doi:10.3389/ti.2026.16314)
Supplement: Supplementary file 3 [file Table1.docx]

**Material and methods, complements**

**Biological dosages**

The lactate deshydrogenase (LDH) was measured in perfusion liquid samples with the CytoTox 96 Non-Radioactive Cytotoxicity Assay (Promega, Madison, USA). Cytokine levels in the perfusion liquid were measured with the Porcine Luminex Discovery Assay to detect TNFα, PECAM, CXCL8, IL-6, IL-10, IL-1α, IL-1β, and IL-1RA (R&D Systems, Biotechne, Minneapolis, USA). Measurements were acquired with a MagPix instrument (Luminex, Austin, TX) and the data were analyzed with the Bio-Plex Manager software (Bio-Rad, Hercules, CA). Carbonylated proteins were quantified from frozen lung tissue samples using a Protein Carbonyl ELISA (OxiSelect^®^, Cell Biolabs Inc, San Diego, USA). Briefly frozen lung tissue pieces were homogenized in 1 mL PBS added with 1 mM EDTA, 25 mM HEPES, 2 % glycerol and ceramic beads 1.4 mm using a Fastprep apparatus (6 m/sec force, 4 times, 4°C). The resulting sample supernatants were collected and the protein concentration was determined using the Pierce ^TM^ BCA Protein assay (ThermoScientific, Rockford, USA). Samples were then normalized to a final protein concentration of 10 µg/mL. Following derivatization with dinitrophenylhydrazine (DNPH), carbonyl groups were detected as DNP adducts using the ELISA kit and quantified against a reference standard. The 8-hydroxy-2'-deoxyguanosine in DNA was quantitated from the same frozen lung tissue samples as above, using a Colorimetric ﻿Universal 8-OHdG ELISA Kit (Novus Biologicals, Biotechne).

**Histology**

﻿The lung samples were embedded in paraffin, and sectioned for hematoxylin and eosin (H&E) staining. The slides were imaged with a slide scanner (Pannoramic SCAN II, v3.0.2, 3DHistech, Medipixel Ltd, Budapest, Hungary). The lesion scores were established based on 5 categories (neutrophils in alveolar spaces, neutrophils in interstitial spaces, hyaline membrane formation, proteinaceous debris, and septal thickening, [1]). Each category was scored on a 0–4 scale. Total scores were averaged across five fields (1.2x1.2 mmm) per sample.

**RNA extraction and quality check**

Total RNA from lung samples was extracted using Trizol and homogenization with 1.4 mm ceramic beads using the Precellys 24 bead grinder homogenizer (Bertin Technologies, St Quentin en Yvelines, France), and purified with the NucleoSpin RNA kit that includes a DNAse digestion step (Macherey-Nagel, Düren, Germany). RNA was quantified using the Qubit RNA assay kit (Thermo Fisher Scientific, Waltham, MA, USA) using the manufacturer’s instructions. RNA was checked for quality with an Agilent 2100 Bioanalyzer using RNA 6000 Nano Kits (Agilent Technologies, Santa Clara, CA, USA). Reverse transcription was conducted on 1.2 µg RNA using the Takara Prime Script RT reagent kit (TaKaRa, Kyoto, Japan) with random primers (5 µM) and oligo dT (2.5 µM) primers. To confirm the absence of genomic DNA contamination, RT-qPCR was performed using RPS24 gene-specific primers (F: AAGGAACGCAAGAACAGAATGAA, R:TTTGCCAGCACCAACGTTG) on RNA samples that had not undergone reverse transcription, using the Q-PCR TB Green Premix ExTaq (TaKaRa).

**Mitochondrial DNA extraction and quantitative PCR**

The DNA was extracted from 1 ml perfusion liquid using the QIAMP® DNA MINI KI (Qiagen, Venlo, Netherland) following the manufacturer instructions. Subsequently, 2 µl of the resulting 50 µl eluate was denaturated at 95°C for 3 min and subjected to qPCR with the Q-PCR TB Green Premix ExTaq (TaKaRa) and the pigND1 primers (F: TTATCTACACCCTAGCAGAAACC, R: AAAGGTCCGGCTGCATATT). A ND1-block was included in order to generate a standard curve for quantitation (ND1 sequence: TAGCCATAATATGATTTATCTACACCCTAGCAGAAACCAACCGAGCCCCGTTCGACCTTACAGAAGGAGAGTCAGAACTTGTATCAGGCTTTAACGTAGAATATGCAGCCGGACCTTTCGCCATATTCTTCATAGCAGA). The data were confirmed using ND4 primers (F: CCCCCATTGCAGGTTCAATA, R : TGCCATAGCCTCCGAGTTTT).

**TAC array design and analysis.**

Gene expression analysis was performed using custom-designed TaqMan® Array Cards (Thermo Fisher Scientific), for 96 gene expression detection, including 4 endogenous controls: RPS24, GAPDH, RPLP2, and HPRT. The gene list (Additional file 2) was established from genes with expression found consistently modulated upon EVLP in our previous work in pig [2] and human [3], with complements from 3 other studies [4; 5; 6]. The cDNA was mixed with TaqMan® Fast Advanced Master Mix and loaded onto the microfluidic cards using a QuantStudio™ OpenArray® AccuFill™ system. The cards were run on a QuantStudio™ 7 Flex Real-Time PCR System. Data were analyzed using the 2^-ΔCt^ method, with normalization to the geometric mean of three housekeeping genes, i.e. RPS24, RPLP2 and GAPDH. HPRT data were too variable across samples based on the Gnorm score of the QuantStudio analysis. Genes with Ct values >32 were considered too weakly expressed and were excluded (IL12B, MC5R, MC1R, MPO, NLRP3, SLADRB1, IL23). The 2^-ΔCT^ values of the 84 remaining genes were used to generate a principal component analysis with the mixOmics (v6.3.1) multi-level package. In the control and WI conditions, differentially expressed genes (DEG) were identified using the oneway_test du package coin [7], using paired data sets between gene expression value at a given timing and time of death. The differential analysis included a Benjamini-Hochberg correction. The DEGs were selected based on an adjusted p-value below 0.1. Expression fold changes (FC) for each gene were calculated relative to the value at the “time of death”. For illustration, the individual fold changes and the mean fold changes (FC) across 5 pigs are shown for the genes that are differentially expressed at least once (69 genes). Finally, the FC values of the DEGs were compared between the different EVLP conditions and the WI 0H (0 hour) condition, using a non-parametric paired test with correction for multiple testing across all conditions. This comparison permitted to count the DEGs for which the FC was significantly different versus the one of WI 0H (adjusted p-value < 0.2) and for which the absolute Log10 mean FC was superior to the one of WI 0H.

**References from Additional file 1**

[1] Y. Zhang, T. Liu, H. Guo, J. Shi, J. Yang, S. Fu, X. Pan, F. Li, H. Zhang, D. Zhang, H. Yang, L. Zheng, M. Shi, and W. Zhou, Ex vivo lung perfusion with GLP-1R agonist mitigates ischemia/reperfusion injury through pyroptosis modulation in lung transplantation- an experimental study. Int J Surg (2025). 10.1097/JS9.0000000000002438

[2] J. De Wolf, C. Gouin, L. Jouneau, M. Glorion, A. Premachandra, F. Pascale, M. Huriet, J. Estephan, J.J. Leplat, G. Egidy, C. Richard, V. Gelin, C. Urien, A. Roux, M. Le Guen, I. Schwartz-Cornil, and E. Sage, Prolonged dialysis during ex vivo lung perfusion promotes inflammatory responses. Front Immunol 15 (2024) 1365964. 10.3389/fimmu.2024.1365964

[3] C. Gouin, T.P. Vu Manh, L. Jouneau, C. Bevilacqua, J. De Wolf, M. Glorion, L. Hannouche, C. Urien, J. Estephan, A. Roux, A. Magnan, M. Le Guen, B. Da Costa, C. Chevalier, D. Descamps, I. Schwartz-Cornil, M. Dalod, and E. Sage, Cell type- and time-dependent biological responses in ex vivo perfused lung grafts. Front Immunol 14 (2023) 1142228. 10.3389/fimmu.2023.1142228

[4] A. Ali, A. Wang, R.V.P. Ribeiro, E.L. Beroncal, C. Baciu, M. Galasso, B. Gomes, A. Mariscal, O. Hough, E. Brambate, E. Abdelnour-Berchtold, V. Michaelsen, Y. Zhang, A. Gazzalle, E. Fan, L. Brochard, J. Yeung, T. Waddell, M. Liu, A.C. Andreazza, S. Keshavjee, and M. Cypel, Static lung storage at 10 degrees C maintains mitochondrial health and preserves donor organ function. Sci Transl Med 13 (2021) eabf7601. 10.1126/scitranslmed.abf7601

[5] C. Baciu, A. Sage, R. Zamel, J. Shin, X.H. Bai, O. Hough, M. Bhat, J.C. Yeung, M. Cypel, S. Keshavjee, and M. Liu, Transcriptomic investigation reveals donor-specific gene signatures in human lung transplants. Eur Respir J 57 (2021). 10.1183/13993003.00327-2020

[6] M. Movahed, S. Brockie, J. Hong, and M.G. Fehlings, Transcriptomic Hallmarks of Ischemia-Reperfusion Injury. Cells 10 (2021). 10.3390/cells10071838

[7] T. Hothorn, K. Hornik, M.A. van de Wiel, and A. Zeileis, A Lego System for Conditional Inference. The American Statistician 60 (2006) 257-263. 10.1198/000313006X118430
